# Spatial Analysis and Spread Monitoring of a Population of *Juniperus macrocarpa* Sm. Across Coastal Dune Systems in Northern Tuscany (Italy)

**DOI:** 10.3390/biology15030278

**Published:** 2026-02-03

**Authors:** Andrea Bertacchi, Diego Orazi, Stefano Bedini, Tiziana Lombardi

**Affiliations:** 1 Department of Agriculture, Food and Environment, University of Pisa, Via del Borghetto, 80, 56124 Pisa, Italy; andrea.bertacchi@unipi.it (A.B.);; 2 Biophysics Institute, National Research Council of Italy (CNR), 56124 Pisa, Italy; 3 Interdepartmental Research Centre for the Study of Climate Change Impacts, University of Pisa, Via del Borghetto 80, 56124 Pisa, Italy

**Keywords:** *Juniperus*, monitoring, dune vegetation, remote sensing, UAV, coastal habitats, Tuscany

## Abstract

**Background**: *Juniperus macrocarpa* Sm. is a key shrub species of the Mediterranean coastal dune systems. The species, considered vulnerable, often shows fragmented or declining populations due to coastal erosion and human pressure. However, along a protected stretch of the northern Tuscany coast it displays an opposite trend, with an apparent expansion of the species. **Methods**: To assess recent population dynamics, we compared high-resolution aerial imagery from 2013 with UAV orthophotos from 2023 across two dune systems of the Migliarino–San Rossore–Massaciuccoli Regional Park (Italy). The dune profile was divided into three belts (B1: shifting dune; B2: consolidated grassland dune; B3: consolidated juniper dune). A total of 368 plots (10 × 10 m) were analyzed to quantify temporal changes in individual abundance and vegetation cover. **Results**: Over the ten-year period, total abundance increased from 99 to 342 individuals (+245%) at Lecciona and from 117 to 324 individuals (+177%) at Marina di Vecchiano. Mean cover per plot increased significantly at both sites (overall *p* < 0.001), with the strongest proportional increases recorded in the seaward belts (B1: up to +1220% in abundance and +4500% in cover) revealing a clear shift from an inner-dune concentration in 2013 to a more homogeneous spatial distribution across the entire dune system in 2023. **Conclusions**: Under conditions of low anthropogenic disturbance, shoreline stability, or geomorphological progradation, *J. macrocarpa* is able to expand well beyond its recognized ecological niche. These findings demonstrate the central role of geomorphological and disturbance regimes in driving coastal dune vegetation dynamics and highlight the need for adaptive, site-specific management strategies for the long-term conservation of priority habitat 2250/EUNIS N1B.

## Simple Summary

This study investigates the recent population dynamics of the shrub *Juniperus macrocarpa* Sm. along a sandy shoreline within a protected area of northern Tuscany (Italy). Changes in population structure were assessed by comparing data collected in 2013 and 2023, integrating remote sensing analyses with field surveys and focusing on individual abundance and plant cover. Results indicate a marked expansion of *J. macrocarpa* from the consolidated inner dune towards the foredune, with particularly strong increases in the shifting dune belt. This pattern reveals an active colonization process, with a higher numerical increase in the seaward belts than in the internal consolidated dunes. Although *J. macrocarpa* is generally considered a late-successional species constrained by strong summer aridity and high insolation in thermo-Mediterranean environments, the meso-Mediterranean coastal conditions of northern Tuscany may mitigate these limiting factors, allowing a wider ecological amplitude and promoting population expansion.

## 1. Introduction

Coastal dune ecosystems are among the most dynamic and vulnerable environments in the Mediterranean region, where natural successional processes interact with intense anthropogenic pressure. Within these systems, woody psammophilous species play a key role in dune stabilization and in the late stages of coastal succession [1].

**Figure 1:**
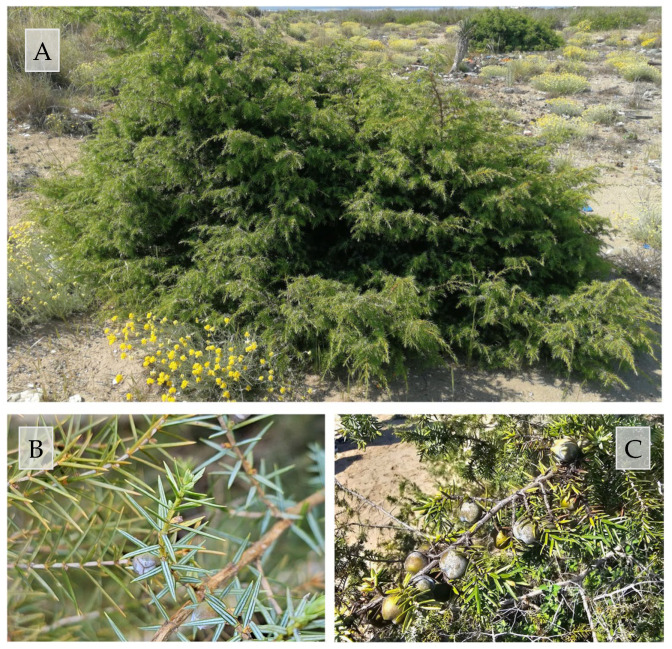
Morphological features of *Juniperus macrocarpa* Sm. (**A**) growth form of adults in the coastal dune environment; (**B**) close-up of leaves and branchlets; (**C**) mature galbuli (reproductive and economic structures).

*Juniperus macrocarpa* Sm. (Figure 1), belonging to the family Cupressaceae (order Pinales), is a diploid species with a chromosome number of 2n = 22. It is a long-lived, evergreen, dioecious conifer characterized by a shrubby habit reaching about 3 m in height, with a Euro-Mediterranean distribution [2]. The species typically inhabits dunes and coastal sand flats and is also present, occasionally, in rocky areas.

From a biological perspective, *J. macrocarpa* is a long-lived, slow-growing conifer characterized by delayed sexual maturity, irregular and generally low seed production, and recruitment occurring mainly through episodic pulses rather than continuous regeneration [3,4]. Seed production is strongly influenced by climatic conditions and exhibits marked interannual variability, while seed dispersal is primarily mediated by birds and small mammals, which play a major role in shaping the spatial and temporal patterns of recruitment [5]. Germination and early seedling establishment are severely constrained by salinity, substrate instability, and summer drought typical of Mediterranean coastal environments; therefore, successful recruitment depends largely on the availability of favorable microsites, where partial substrate stabilization and reduced environmental stress facilitate seedling survival and early growth [6,7].

*J. macrocarpa* is a diagnostic, constant, and dominant species of EUNIS habitat N1B “Mediterranean and Black Sea coastal dune scrub spp.” [8], corresponding to the priority habitat 2250 “Coastal dunes with *Juniperus* spp.” of the EU Habitats Directive (92/43/EEC). This habitat, characterized by juniper-dominated scrub formations, represents the most mature stage of psammophilous succession on coastal sand dunes [9].

Across the Mediterranean Basin, *J. macrocarpa* occupies a range of coastal habitats, including embryonic dunes, fixed dunes, and stabilized inner dune systems. Its distribution and population dynamics are strongly influenced by geomorphological processes, disturbance regimes, and human pressures. In several Mediterranean regions, including southern Spain, Sardinia, Sicily, Corsica, and other central Mediterranean coasts, populations are frequently reported as fragmented, with limited recruitment and high sensitivity to both natural disturbances (storm surges, erosion, prolonged drought) and anthropogenic impacts such as coastal development, tourism infrastructure, and trampling [4,10].

However, although Italy hosts the main area of this habitat at the EU level followed by Spain and France [11], the distribution in Italy of *J. macrocarpa* is highly discontinuous, with large habitat remnants mainly confined to Sardinia, Tuscany, Latium, and Apulia [12].

In Tuscany, habitat N1B is formally identified within the various protected areas that are part of the Natura 2000 network [13], for a total coastal development of about 50 km.

Although phytocoenoses of *J. macrocarpa* are still distributed in almost all the sandy coasts of the region, numerous critical factors such as access to the sea, bathing establishments, summer sports facilities, roads, ports, and coastal erosion severely limit their diffusion [14,15,16,17]. Within the N1B habitat, *J. macrocarpa* is normally widely distributed in the innermost belt of the consolidated dune. Furthermore, it colonizes stabilized coastal dune grasslands (N16 EUNIS habitat) up to the edge of the mobile dunes with *Ammophila arenaria* (N13 EUNIS habitat) [2,8,18]. Along the Tuscan coast, this distribution dynamic is present in almost all the coastal sectors [19,20,21,22,23,24,25].

Despite the extensive literature describing the ecology and conservation status of *Juniperus macrocarpa*, important knowledge gaps remain regarding its long-term population dynamics and the mechanisms driving its contrasting trends across Mediterranean coasts. In particular, few studies have provided quantitative, spatially explicit assessments of decadal-scale changes in both population structure and spatial organization of this species within coastal dune systems. Moreover, the apparent expansion of *J. macrocarpa* observed along parts of the Tuscan coast contrasts sharply with the widespread decline reported for most Mediterranean regions, and the ecological and geomorphological drivers of this divergence remain poorly understood [4,10]. This lack of integrative, site-comparative analyses currently limits the ability to predict future trajectories of this priority habitat under changing environmental and management conditions.

Recently, a notable expansion of the species has been documented in two coastal sites located north of the Arno River (Migliarino–San Rossore–Massaciuccoli Regional Park, Tuscany, Italy) [26]. This trend contrasts with the southern sectors of the park, where habitat N1B has undergone severe regression and fragmentation due to coastal erosion [15,27,28].

The present study aims to characterize the spatial distribution and recent dynamics of *Juniperus macrocarpa* populations along this sector of the coast by comparing two discrete time points (2013 and 2023), thereby allowing the assessment of decadal-scale changes in population structure, spatial organization, and dune system dynamics.

In particular, the extent and spatial patterns of *J. macrocarpa* expansion were quantified across the dune system with reference to the main vegetation belts that typify Mediterranean psammophilous zonation.

This approach was implemented through integrated field monitoring and the analysis of high-resolution satellite imagery (2013) and UAV-based orthophotos (2023).

Current approaches to vegetation mapping increasingly rely on the combined use of field surveys and remote sensing techniques, with a growing application of UAV platforms [29,30,31]. The use of UAV technology, providing low-altitude and very high-resolution imagery, allows not only the identification and mapping of phytocoenoses but also the detection of plant species at the individual level [32,33,34].

By integrating remote sensing analyses with detailed ground-based surveys, robust estimates of plant cover, individual density, and spatial organization along the sea–inland gradient were obtained. Beyond a purely descriptive assessment, this approach also supports an ecological interpretation of the observed distribution patterns, allowing evaluation of whether the current expansion of *J. macrocarpa* reflects natural successional dynamics and enhanced stabilization of dune systems.

From this perspective, the results of this study contribute to a better understanding of the functioning and evolution of Mediterranean coastal dune ecosystems and provide a spatially explicit baseline to support long-term monitoring, conservation planning, and adaptive management strategies for priority habitat N1B.

## 2. Materials and Methods

### 2.1. Study Area

**Figure 2:**
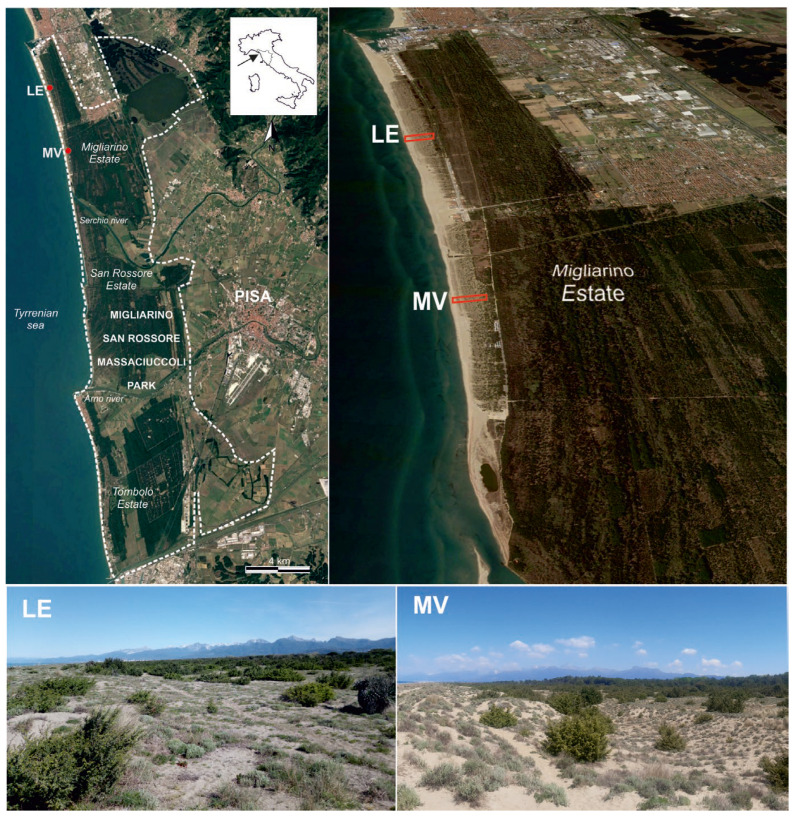
Study area within the Migliarino–San Rossore–Massaciuccoli Regional Park (Tuscany, Italy). The upper panels show the geographical setting of the park and the location of the two investigated coastal sites (LE = Lecciona; MV = Marina di Vecchiano). The lower panels provide ground-level views of the dune landscapes at both sites, illustrating the geomorphological context and the spatial structure of the coastal vegetation.

The investigated areas are part of the coast of the Migliarino San Rossore Massaciuccoli Park (Tuscany, Italy) (between 43°51′36″–43°35′25″ N and 10°14′26″–10°21′11″ E) (Figure 2). In 2004, the park was designated by UNESCO a Biosphere Reserve [35].

Although accounting for only a small fraction of the park area (1.7% of the total), the dune system represents an environment of high ecological value in terms of coastal habitat diversity [27]. In particular, the study area is located in the northern sector of the park (Tenuta di Migliarino), comprising about 8 km of coast subject to protective restrictions but freely accessible to the public. Since the 1950s, the coastline has progressed steadily until it stabilized in 2019. On the contrary, the southern sector of the park is under an imposing erosive process (Tenuta di San Rossore) [36] or partly transformed by bathing establishments (Tenuta di Tombolo) [28].

To characterize the local climate, temperature and precipitation data for the 2013–2023 interval were obtained from the weather station of Lido di Camaiore (Lucca, Tuscany; SIR code TOS11000011; 43.898° N, 10.243° E), located approximately 7–11 km north of the study sites [37].

The study area shows a mean annual temperature of 15.4 °C, with thermal peaks occurring in July (mean monthly maximum: 23.8 °C). The annual average rainfall for the investigated decade was 1090 mm, with a peak recorded in November (170 mm).

**Figure 3:**
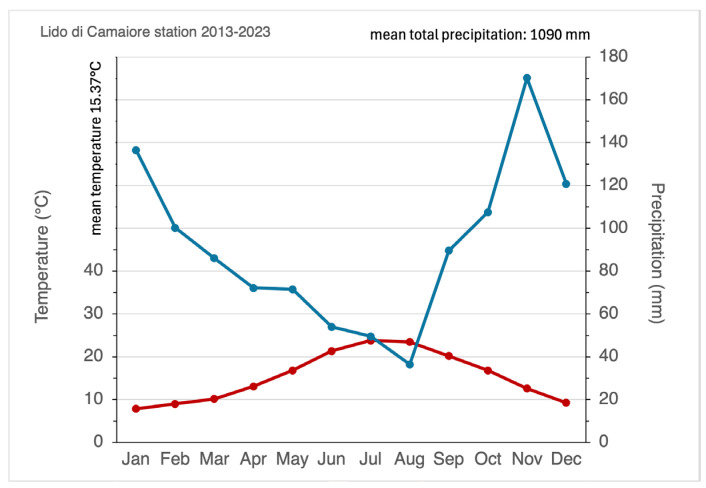
Walter–Lieth climate diagram for the Lido di Camaiore weather station. Data refer to the 2013–2023 period. The red line represents mean monthly temperature (°C), while the blue line represents total monthly precipitation (mm). The dotted/yellow area indicates the period of seasonal aridity (summer drought), where precipitation falls below the aridity threshold (P < 2T). Mean annual temperature (15.4 °C) and total annual precipitation (1090 mm) are reported at the top.

According to the Walter–Lieth diagram (Figure 3), the area exhibits a typical Mediterranean seasonality with a distinct period of summer drought. While strictly defined aridity (P < 2T) is most pronounced in August, water shortages frequently extend from June to September due to high evaporation rates and irregular rainfall patterns.

Indeed, beyond average values, the precipitation regime is characterized by pronounced temporal irregularity. During the summer quarter (June–August), the mean frequency of rainfall events is extremely low, averaging only 4.0, 2.9, and 3.5 rainy days per month, respectively. These long sequences of consecutive rainless days, combined with high summer temperatures and strong coastal winds, generate severe water stress for dune vegetation during the critical growing season.

Interannual variability further amplifies these constraints, producing alternating years of severe water deficit and short periods of higher moisture availability.

According to the bioclimatic classification of Pesaresi et al. [38], the study area falls within the Mediterranean macrobioclimate, with an upper meso-Mediterranean thermotype and a lower subhumid ombrotype. This climatic framework defines a highly selective environment for coastal dune vegetation, where plant recruitment and survival are strongly controlled by the interaction between episodic rainfall pulses and prolonged summer drought.

### 2.2. Remote Sensing and GIS

Along this stretch of coast, two rectangular areas orthogonal to the coastline have been selected: Lecciona (LE: 43.831205° N, 10.252566° E) and Marina di Vecchiano (MV: 43.804642° N, 10.263243° E) (Figure 2).

This choice was based on the extent of N1B habitat and the consistency of the dune environment. The two areas have a width of 80 m and comparable depths (282 m for Lecciona, 315 m for Marina di Vecchiano). The inner edge was represented by the substitution of N1B habitat with strict woodlands habitats.

The investigation was designed as a diachronic study comparing the spatial structure and population dynamics of *J. macrocarpa* in the years 2013 and 2023.

Two different orthophoto datasets were used to construct the experimental design and perform spatial analyses and processed in a GIS environment (QGIS v.3.4) [39]. The first is a regional aerial survey from October 2013 [40] with a resolution of 20 cm/pixel. The second was acquired in May 2023 using a DJI Mini 3 Pro drone (SZ DJI Technology Co., Ltd., Shenzhen, China).

The UAV was equipped with a standard RGB camera (1/1.3-inch sensor, 24 mm equivalent focal length, f/1.7 aperture, FOV 82.1°, SZ DJI Technology Co., Ltd., Shenzhen, China) capturing 48 MP images (4032 × 3024 pixels). Flight parameters included a shutter speed of 1/1600 s and ISO 110. A total of 185 and 236 nadir images were collected for Lecciona and Marina di Vecchiano, respectively, at a flight altitude of 25 m AGL and a speed of 2.5 m/s (forward overlap: 60%; side overlap: 80%). This resulted in a Ground Sampling Distance (GSD) of approximately 0.5 cm/pixel.

An orthomosiac was performed using the Agisoft Metashape 17.5, achieving an RMSE (root mean square error) reprojection of 0.29 pixels.

The orthomosaic data from 2013 were georeferenced in WGS 84/UTM zone 32N (EPSG: 32632). In the QGIS environment, UAV images were automatically overlaid on orthomosaic 2013. The observed average shift between datasets was approximately 20 cm. This alignment error was considered negligible for the identification and positioning of *J. macrocarpa* individuals.

**Figure 4:**
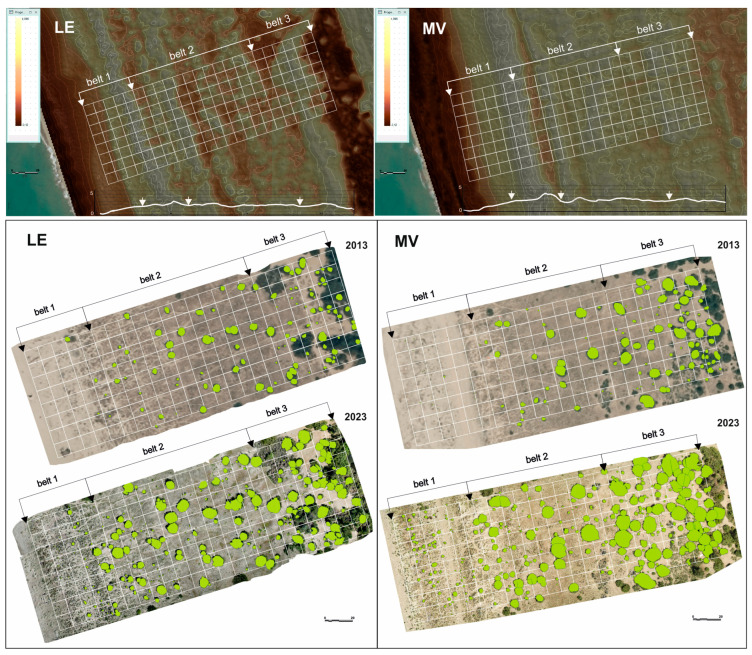
Sampling grid and dune profiles overlaid on the regional digital terrain model (DTM) (upper panels); orthophotomosaics of the two study sites in 2013 and 2023 with *Juniperus macrocarpa* canopy cover mapped (lower panels). LE = Lecciona; MV = Marina di Vecchiano.

A 10 × 10 m grid was overlaid on each study area, dividing it in three dune belts: B1 (drift lines and shifting dunes, EUNIS N12–N14), B2 (consolidated grassland dunes, EUNIS N16), and B3 (coastal dune scrub, EUNIS N1B). A total of 184 plots were analyzed per site. However, the distribution among bands varied due to the different habitat depths: Lecciona comprised 40 plots in B1, 96 in B2, and 48 in B3, while Marina di Vecchiano included 48 plots in B1, 80 in B2, and 56 in B3. This difference in the number of plots was due to the different depths of the habitats in the two areas (Figure 4).

Finally, the crowns of *J. macrocarpa* individuals were identified through manual photointerpretation and subsequently traced by GIS vector tools, considering all individuals wholly or partially within the plots with a minimum diameter of 30 cm (a threshold dictated by the lower resolution of the 2013 dataset).

Field surveys were subsequently carried out to validate the accuracy of photointerpretation and identification of the investigated species.

Kernel density estimation (KDE) was applied to georeferenced *J. macrocarpa* occurrence data to generate continuous density surfaces describing spatial patterns across the dune system. Using a Gaussian kernel function, KDE allowed the identification of density gradients and aggregation zones along the sea–inland transect. Bandwidth selection was optimized to capture spatial structure while minimizing noise. Density surfaces were normalized to compare distribution patterns between survey years and among dune vegetation belts [41].

### 2.3. Statistica Analysis

*J. macrocarpa* population data were analyzed using univariate analyses of variance (ANOVA), with the number of individuals, coverage area per plot, and coverage area per individual as dependent variables, and year (2013, 2023), belt (B1, B2, B3), and site (Lecciona, Marina di Vecchiano) as fixed factors. The assumption of homogeneity of variances was tested using Levene’s test. When this assumption was not met, Tamhane’s T2 post hoc test was applied for multiple comparisons. All statistical analyses were performed using SPSS Statistics 26.0 (IBM Corp., Armonk, NY, USA).

## 3. Results

### 3.1. Juniperus macrocarpa Plant Density

The statistical analyses of the *J. macrocarpa* population data showed that the total number of individuals was overall not different between the two sites (*F* = 0.143, *p* = 0.706) but varied significantly among the three belts (*F* = 20.978, *p* < 0.001) and between years (*F* = 221.751, *p* < 0.001). Crucially, a significant Belt × Year interaction was observed (*F* = 4.371, *p* = 0.013), indicating that temporal changes in density were not uniform across the dune profile.

Over the decade, the total number of individuals increased markedly, from 99 to 342 (+245%) at Lecciona and from 117 to 324 (+177%) at Marina di Vecchiano with significant increases both at Lecciona (*F* = 114.244, *p* < 0.001) and Marina di Vecchiano (*F* = 127.562, *p* < 0.001) site.

**Figure 5:**
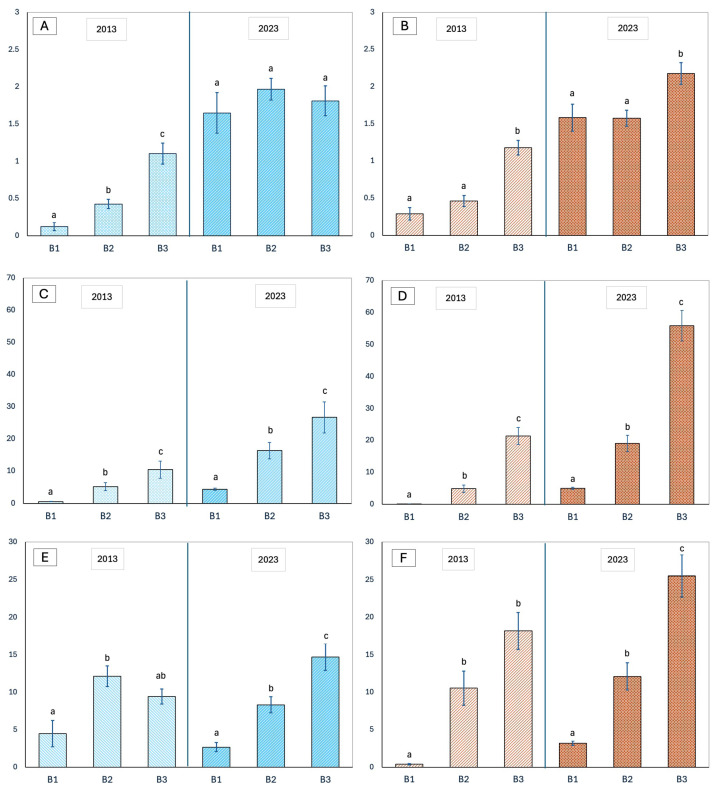
Population structure and temporal dynamics of *Juniperus macrocarpa* at the Lecciona (LE) and Marina di Vecchiano (MV) sites in 2013 and 2023 along the sea–inland dune gradient. (**A**,**B**) Mean number of individuals per plot at Lecciona and Marina di Vecchiano, respectively. (**C**,**D**) Mean *J. macrocarpa* cover per plot at Lecciona and Marina di Vecchiano, respectively. (**E**,**F**) Mean cover per individual at Lecciona and Marina di Vecchiano, respectively. Data are shown for the three vegetation belts (B1–B3). Error bars represent standard errors. Different letters indicate statistically significant differences according to Tamhane’s T2 post hoc test.

At Lecciona, the number of *J. macrocarpa* individuals increased significantly from 2013 to 2023 (*F* = 114.244, *p* < 0.001). In 2013, the mean number of individuals per plot increased progressively from the seaward to the inland belt, with values ranging from 0.13 ± 0.05 in B1 to 0.43 ± 0.06 in B2 and 1.10 ± 0.14 in B3 with significant differences among belts (*F* = 25.707, *p* < 0.001). These values corresponded to 5, 41, and 53 individuals, respectively. In 2023, all belts showed higher values than in 2013. B1 increased to 1.65 ± 0.27 (66 individuals) and B2 to 1.97 ± 0.15 (189 individuals), corresponding to absolute increases of +1.52 and +1.54 individuals per plot (equivalent to +1220% and +361%, respectively). B3, which already supported higher densities in 2013, showed a more moderate increase, reaching 1.81 ± 0.20 (53 individuals), corresponding to an absolute change of +0.71 and a proportional increase of +64%. Overall, in 2023, the differences among belts were less pronounced than the ones in 2013 and statistically not significant (*F* = 0.671, *p* = 0.512), resulting in a statistical homogenization of the distribution along the B1–B3 zonation sequence (Figure 5A).

Consistent with findings at Lecciona, at Marina di Vecchiano the number of *J. macrocarpa* individuals also increased significantly from 2013 to 2023 (*F* = 116.437, *p* < 0.001). In 2013, the site showed a trend similar to Lecciona, with significant differences among belts (*F* = 25.277, *p* < 0.001) and mean abundances ranging from 0.29 ± 0.08 in B1 to 0.46 ± 0.08 in B2 and 1.18 ± 0.10 in B3, corresponding to 14, 37, and 66 individuals, respectively. In 2023, all belts showed higher abundances. B1 and B2 both reached 1.58 ± 0.18 and 1.58 ± 0.11 (76 and 126 individuals), while B3 reached 2.18 ± 0.15 (122 individuals). Although absolute increases were comparable across belts (+1.29, +1.11, and +1.00 individuals per plot for B1, B2 and B3), proportional increases differed appreciably, with B1 rising by +445%, B2 by +243%, and B3 by +85%. Crucially, in contrast to observations at Lecciona, the original spatial structure at Marina di Vecchiano was preserved, with significant differences among belts (*F* = 5.956, *p* = 0.003) and B3 remaining the belt with the highest densities (Figure 5B).

### 3.2. Juniperus macrocarpa Cover per Plot

The *J. macrocarpa* cover per plot was, overall, significantly different between the two sites (*F* = 31.340, *p* < 0.001) with significant differences among the three belts (*F* = 128.312, *p* < 0.001) and between years (*F* = 123.886, *p* < 0.001), with a significant belt × year interaction (*F* = 25.370, *p* < 0.001).

Across both study sites, mean cover per plot increased along the B1–B3 zonation sequence in 2013, and higher values were recorded in all belts in 2023. The magnitude of increase, however, differed among belts and between sites.

At Lecciona, in 2013, mean cover per plot increased significantly from the seaward belt toward the inland belt (*F* = 21.683, *p* < 0.001), ranging from 0.56 ± 0.25 m^2^ in B1 to 10.47 ± 7.00 m^2^ in B3. In 2023, cover values were significantly higher than in 2013 (*F* = 61.262, *p* < 0.001) and a significant difference among belts was maintained (*F* = 22.565, *p* < 0.001). B1 increased to 4.42 ± 0.93 m^2^, corresponding to an absolute increase of +3.86 and a proportional increase of +689%. B2 rose from 5.18 to 16.38 ± 1.81 m^2^ (+216%), while B3 increased from 10.47 to 26.69 ± 2.33 m^2^ (+155%). Although B3 remained the belt with the highest absolute cover in both years, the proportional increase highlights a marked rise in cover in the most seaward belt (B1) (Figure 5C).

Consistent with findings at Lecciona, at Marina di Vecchiano mean cover per plot also increased along the B1–B3 zonation sequence in 2013, ranging from 0.11 ± 0.04 m^2^ in B1 to 21.42 ± 2.67 m^2^ in B3, with significant differences among belts (*F* = 40.415, *p* < 0.001) reflecting a strong gradient in vegetation structure. In 2023, all belts exhibited marked increases. B1 increased from 0.11 to 5.06 ± 0.33 m^2^ (+4500%), representing the largest proportional change observed across both sites. B2 rose from 4.87 to 19.08 ± 2.57 m^2^ (+292%) while B3 showed the highest absolute cover, increasing from 21.42 to 55.84 ± 4.78 m^2^ (+161%). Despite these marked increases, the original gradient pattern was retained, with B3 consistently supporting the highest cover values (*F* = 60.182, *p* < 0.001) (Figure 5D).

### 3.3. Mean Cover per Individual

Patterns of mean cover per individual showed spatial variation along the B1–B3 zonation sequence in both survey years, with distinct temporal trajectories at the two study sites. While some belts exhibited decreases, others showed marked increases over time. Statistics showed that coverage per individual was significantly different among belts (*F* = 18.182, *p* < 0.001), but did not differ between years (*F* = 0.975, *p* = 0.324) or between sites (*F* = 2.473, *p* = 0.116). However, significant site × belt (*F* = 5.087, *p* = 0.006) and belt × year (*F* = 3.044, *p* = 0.048) interactions were detected.

At Lecciona, the *J. macrocarpa* cover per individual showed no significant difference between the two years (*F* = 0.897, *p* = 0.344) but significant differences among the three belts (*F* = 13.715, *p* < 0.001). In 2013, no significant differences were detected among belts (*F* = 2.783, *p* = 0.067) with a mean cover per individual that ranged from 4.47 ± 1.76 m^2^ in B1 to 12.14 ± 1.39 m^2^ in B2, decreasing slightly in B3 (9.44 ± 1.00 m^2^). By contrast, in 2023, significant differences among belts emerged (*F* = 22.565, *p* < 0.001). B1 and B2 showed lower values compared to 2013, with mean cover decreasing to 2.68 ± 0.62 m^2^ in B1 (−40.06%) and to 8.32 ± 1.07 m^2^ in B2 (−31.46%). Conversely, mean cover increased in B3, rising to 14.73 ± 1.80 m^2^ (+55.97%) (Figure 5E).

At Marina di Vecchiano, similar to Lecciona, there was no significant difference between years (*F* = 0.333, *p* = 0.564) but a strong gradient among belts (*F* = 28.632, *p* < 0.001). In 2013, mean cover per individual increased significantly along the B1–B3 zonation sequence (*F* = 7.665, *p* < 0.001) ranging from 0.39 ± 0.09 m^2^ in B1 to 10.54 ± 2.29 m^2^ in B2 and 18.17 ± 2.45 m^2^ in B3. The differences among belts were also mainteined in 2023 (*F* = 23.524, *p* < 0.001), even if all belts exhibited higher values with respect to 2013. B1 values increased to 3.20 ± 0.30 m^2^ (+714.69%), B2 to 12.11 ± 1.82 (+14.95%), and B3 to 25.50 ± 2.84 m^2^ (+40.36%) (Figure 5F).

### 3.4. Kernel Density Estimation

**Figure 6:**
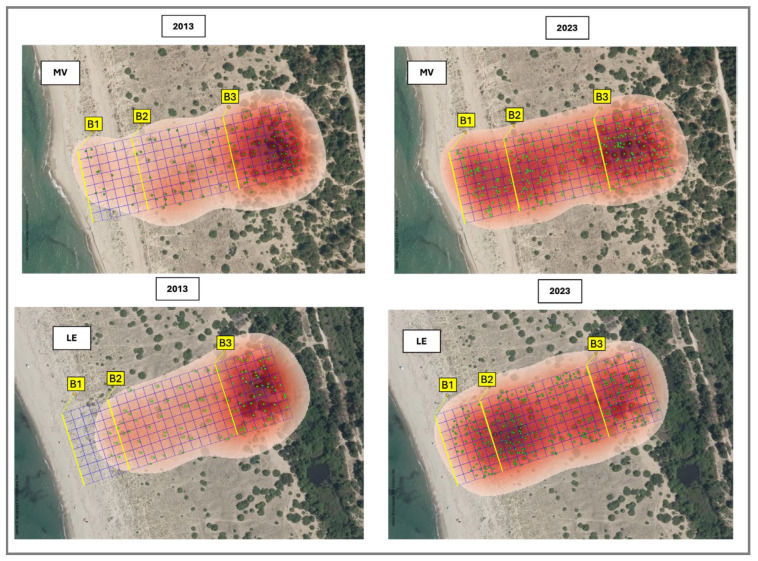
Kernel density estimation (KDE) of *Juniperus macrocarpa* individuals at Marina di Vecchiano (MV) and Lecciona (LE) in 2013 and 2023. Density surfaces illustrate changes in spatial aggregation patterns across the three dune belts (B1–B3) along the sea–inland gradient.

Kernel density estimation (KDE) was applied to evaluate the spatial distribution and aggregation patterns of *J. macrocarpa* individuals in 2013 and in 2023. The analysis cofirms the ANOVA results, revealing a similar spatial configuration of the two sites and a significant temporal shift in population structure. The comparison between the kernel density surfaces and the hotspot distributions for 2013 and 2023 reveals a clear temporal shift in the spatial organization of the *J. macrocarpa* population along the sea–inland gradient. In 2013, individuals were predominantly clustered within belt B3, corresponding to the consolidated dune, while only sporadic pioneer specimens occurred in belts B2 and B1, closer to the foredune and embryonic dune areas. By 2023, however, the density maps display a more homogeneous distribution throughout the dune profile, characterized by the emergence of distinct high-density hotspots across all three belts (B1, B2, and B3) (Figure 6).

## 4. Discussion

This study documents a pronounced expansion of *J. macrocarpa* within the analyzed coastal dune systems, characterized by substantial increases in abundance, cover, and spatial extent. In contrast to the widespread decline reported for Mediterranean dunes, the observed patterns highlight a rapid colonization of shifting dune belts and a progressive shift toward a more homogeneous distribution across the dune profile.

The distribution of *J. macrocarpa* along the sea–inland gradient reflects the strong influence of abiotic constraints in structuring Mediterranean dune vegetation, operating in close interaction with biotic processes, including species interactions and facilitation mechanisms [7].

Pronounced differences in the *J. macrocarpa* abundance were observed among belts across both sites and years, with B3 generally supporting the highest values, whereas patterns of mean individual size varied by site and year along the landward–seaward gradient [1]. Consistent with findings from other Mediterranean coastal systems [2,10], *J. macrocarpa* reaches its ecological optimum in the inner dune belt (B3). This belt, characterized by greater substrate stability and water availability, traditionally supports the highest density of large, established individuals. Conversely, in accordance with the Stress Gradient Hypothesis [42], the seaward belt (B1) represents a physiological threshold, where intense salt spray, wind exposure, burial, and abrasion impose strong constraints on adult growth and survival, while potentially enhancing the relative importance of facilitative interactions during early life stages.

The comparison between *J. macrocarpa* populations in 2013 and 2023 revealed a significant overall increase in the number of individuals across all belts and at both sites, particularly in B1.

However, changes in abundance were not always accompanied by proportional changes in individual size or total cover, indicating a partial decoupling between demographic expansion and structural development of the shrub layer.

While overall cover provides an integrated measure of population expansion and habitat occupation, additional information on individual canopy size and fine-scale spatial arrangement would further improve the interpretation of population structure and dispersal dynamics. Nevertheless, the strong and consistent increase in cover observed across belts and years clearly documents a major expansion of *J. macrocarpa* within the dune system.

Although plot-based metrics provide a robust measure of macro-scale demographic expansion, the discrepancy between density and cover highlights the need for finer-scale spatial analysis. Future studies incorporating nearest-neighbor analyses could further elucidate the specific facilitative interactions that are likely enabling this colonization in high-stress zones. Despite the environmental severity of the foredune, the observed pattern suggests two complementary ecological drivers. First, density-dependent regulation appears to limit further expansion in the inner dune belt (B3); here, large adult individuals occupy extensive areas (up to ~25 m^2^), generating strong competition for light and space that limits seedling establishment (self-thinning). Second, microsite availability in the shifting dune belts (B1 and B2) drives the current expansion; the open and discontinuous vegetation structure provides abundant gaps where reduced intraspecific competition facilitates seed germination and early survival [43], effectively channeling the recent mass recruitment event toward the sea.

Consequently, the marked increase in abundance in B1 in 2023 represents a clear colonization event rather than a regenerative phase. Unlike the inner belts, where recruitment is often driven by gap formation following adult mortality, the expansion in B1 was likely promoted by the high availability of open, competition-free microsites. This condition allowed the establishment of pioneer individuals despite the harsh environmental conditions, consistent with the episodic recruitment pulses typical of long-lived species in shifting dune systems [5,6]. The absence of a significant concomitant temporal increase in mean individual cover further supports the interpretation that populations in B1 are dominated by young or subadult individuals that have not yet contributed substantially to biomass accumulation.

Collectively, data from the two study sites indicate positive demographic trajectories, unlike much of the rest of the sandy coasts of Tuscany [15,19,25] and, more generally, of the Mediterranean area, where *J. macrocarpa* is often reported as vulnerable [4,10]. This pattern is likely related not only to the low level of human disturbance but also to the long-term progradation of the coastline and the specific geomorphological configuration of this sector of dune systems. Here, dunes are characterized by a low-relief and weakly articulated morphology, resulting in broad and relatively uniform surfaces that favor progressive colonization and stabilization.

The significant Site × Belt interaction both for *J. macrocarpa* plant density, and cover per plot, reveals distinct ecological dynamics at the two dune systems, suggesting important site-specific differences that cannot be fully explained by a simple sea–inland successional model.

At Lecciona, large individuals were mainly concentrated in belt B2 in 2013 and maintained a similar spatial configuration in 2023, suggesting a relatively homogeneous and already stabilized system across much of the gradient.

In contrast, at Marina di Vecchiano, the spatial distribution of individual size follows a more orderly and pronounced sea–inland gradient, with larger and more developed shrubs increasingly concentrated in the inner dune belt (B3). This pattern is consistent with a classical successional trajectory of Mediterranean dune systems, in which structural maturity and biomass accumulation progressively increase from the foredune toward the stabilized inner dune.

These contrasting patterns suggest that present-day population structure is not controlled solely by current environmental gradients, but is strongly shaped by the geomorphological history of each site and by processes of historical contingency, which collectively determine long-term dune stabilization and vegetation dynamics.

At Marina di Vecchiano, the simultaneous increase in abundance and size of individuals reflects a structurally coherent trend of dune stabilization or progradation.

At Lecciona, the pronounced decoupling between increasing density and decreasing individual size in the B1 belt likely reflects a recent phase of colonization, although the influence of episodic disturbance cannot be excluded. Indeed, although erosion is absent at both sites—a rare feature along Mediterranean coasts—the foredune remains highly exposed to mechanical stress caused by storm surges, salt spray, and strong winds, all factors known to induce canopy regression in coastal juniper stands [37,44].

The resulting creation of colonizable gaps likely activated the seed bank and promoted the massive recruitment observed in 2023. This mechanism aligns with the well-documented disturbance–recovery dynamics typical of shrub species in variable dune environments [6].

In both sites, the inner dune belt (B3) emerges as the primary resilience reservoir of the dune system. Large individuals within this belt maintain structural continuity, accumulate substantial biomass, and ensure long-term propagule production. The increase in mean individual size observed between 2013 and 2023 suggests that the carrying capacity for *J. macrocarpa* in the inner dune has not yet been reached, thereby allowing further densification and structural consolidation of the shrub layer. Similar dynamics have been reported in other Mediterranean shrub communities undergoing late-successional consolidation [45].

Accordingly, the inner dune fulfills a dual ecological function: (i) it acts as a key structural stabilizer of the system, and (ii) it serves as a propagule source facilitating the recolonizing of more vulnerable outer dune belts.

The decade-long trends observed here allow cautious predictions of future system dynamics. A key feature distinguishing these sites from many Mediterranean dune systems is the absence of active coastline erosion, a condition that may enhance long-term vegetation stability and promote the continued expansion of *J. macrocarpa* towards the outer dune belts.

Under scenarios of reduced anthropogenic pressure, geomorphological stability, and adequate sediment supply, B1 and B2 belts are expected to undergo further *J. macrocarpa* population densification. At Lecciona, where the zonation gradient is already weakened, this process may lead to a progressive reduction in habitat heterogeneity, with increasing dominance of *J. macrocarpa* and a consequent loss of distinct zonation between early- and late-successional dune belts. At Marina di Vecchiano, future dynamics are more likely to involve the coalescence of shrub nuclei within the B2–B3 belts, resulting in increasingly continuous juniper stands. Nevertheless, even in the absence of chronic erosion, rising climatic variability—including extreme storm events, intensified wind regimes, and prolonged drought—may induce episodic mortality and trigger disturbance–recovery cycles, as widely reported for Mediterranean dunes ecosystems [46,47].

Overall, in the absence of active management, *J. macrocarpa* is expected to continue its spatial expansion, reinforcing dune stabilization but potentially constraining early-successional open habitats. Long-term monitoring, coupled with scenario-based management strategies, will therefore be crucial to balance conservation objectives with ongoing natural successional dynamics.

## 5. Conclusions

Overall, the results of this study demonstrate that *J. macrocarpa* populations in both investigated sites are in good demographic condition and are undergoing a marked phase of spatial expansion. However, the observed dynamics are not uniform across sites and reflect the combined influence of geomorphological history, local disturbance regimes, and current environmental constraints.

At Marina di Vecchiano, *J. macrocarpa* population growth follows a coherent successional trajectory, with increasing abundance and canopy development converging toward the inner dune belt (B3), consistent with progressive dune stabilization and limited recent disturbance. At Lecciona, the population exhibits a more homogeneous spatial structure along the sea–inland gradient, shaped by earlier stabilization processes and by the legacy of past geomorphological conditions. This has resulted in site-specific internal organization and a partial decoupling between density and individual size, particularly in the foredune belt.

The pronounced expansion of *J. macrocarpa* observed along this stretch of Tuscan coast appears to be driven by a unique combination of favorable factors, including long-term geomorphological stability, the absence of chronic shoreline erosion, reduced anthropogenic pressure within protected areas, and a climatic regime characterized by strong inter-annual variability and episodic stress. Together, these conditions promote both adult persistence and repeated recruitment pulse, allowing the species to progressively colonize seaward dune sectors. This combination of drivers helps explain why similar expansion patterns are not consistently observed in many other European coastal regions, where *J. macrocarpa* occurs but remains more fragmented or demographically constrained due to stronger erosion, higher human pressure, or less favorable disturbance regimes.

From a conservation perspective, these findings provide concrete guidance for the management of Mediterranean coastal dune ecosystems. The identification of site-specific demographic trajectories highlights the need for locally adapted conservation strategies rather than uniform management prescriptions. In areas characterized by strong *J. macrocarpa* expansion and geomorphological stability, management actions should aim to preserve dune continuity while maintaining a mosaic of early- and late-successional habitats. Conversely, in sectors subject to erosion or higher anthropogenic pressure, conservation priorities should focus on protecting remnant juniper stands, enhancing natural regeneration processes, and limiting physical disturbance.

The spatial baseline provided here enables repeated UAV-based monitoring to track recruitment, canopy growth, and disturbance–recovery cycles, supporting early-warning detection of shifts in dune zonation under increasing climatic variability. Future work integrating individual-level spatial metrics (e.g., nearest-neighbor analyses) will refine mechanistic interpretations of facilitation and dispersal processes and improve adaptive management of habitat 2250/EUNIS N1B.

## Data Availability

The data presented in this study are available from the corresponding author upon reasonable request.
